# The Protective Role of 4-Acetylarylquinolinol B in Different Pathological Processes

**DOI:** 10.3390/cimb44050161

**Published:** 2022-05-23

**Authors:** Huijie Zhao, Huiyang Liu, Yihan Yang, Honggang Wang

**Affiliations:** 1 Institute of Chronic Disease Risks Assessment, Henan University, Kaifeng 475004, China; zhj5696@163.com; 2 School of Basic Medical Sciences, Henan University, Kaifeng 475004, China; m15736875597@163.com (H.L.); h1323240458@163.com (Y.Y.)

**Keywords:** 4-acetylarylquinolinol B, hepatocellular carcinoma, glioblastoma, osteoclastogenesis, nonalcoholic fatty liver diseases

## Abstract

Antrodia cinnamomea is a traditional plant and a unique fungus native to Taiwan that has been reported to have many biological functions, including anti-inflammatory and anticancer activities. The compound 4-acetylarylquinolinol B (4-AAQB) is one of the main bioactive compounds in the stamens of Antrodia cinnamomea, and has many biological functions, such as anti-inflammatory, antiproliferative, blood sugar reduction, antimetastasis, and vascular tone relaxation. In recent years, the increasing evidences have shown that 4-AAQB is involved in many diseases; however, the relevant mechanisms have not been fully clarified. This review aimed to clarify the improvement by 4-AAQB in different pathological processes, as well as the compound’s molecular mechanisms, in order to provide a theoretical reference for future related research

## 1. Introduction

**Figure 1:**
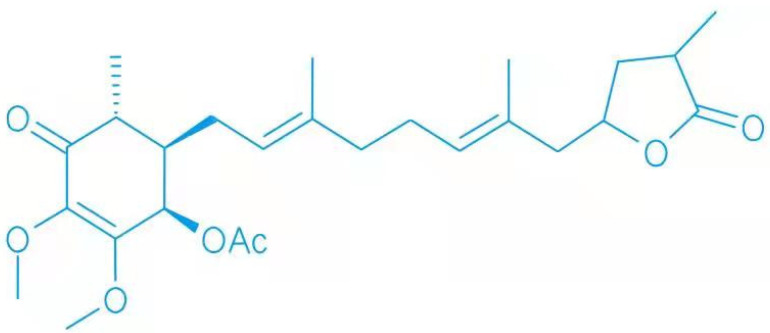
The chemical structure of 4-acetylarylquinolinol B.

**Figure 2:**
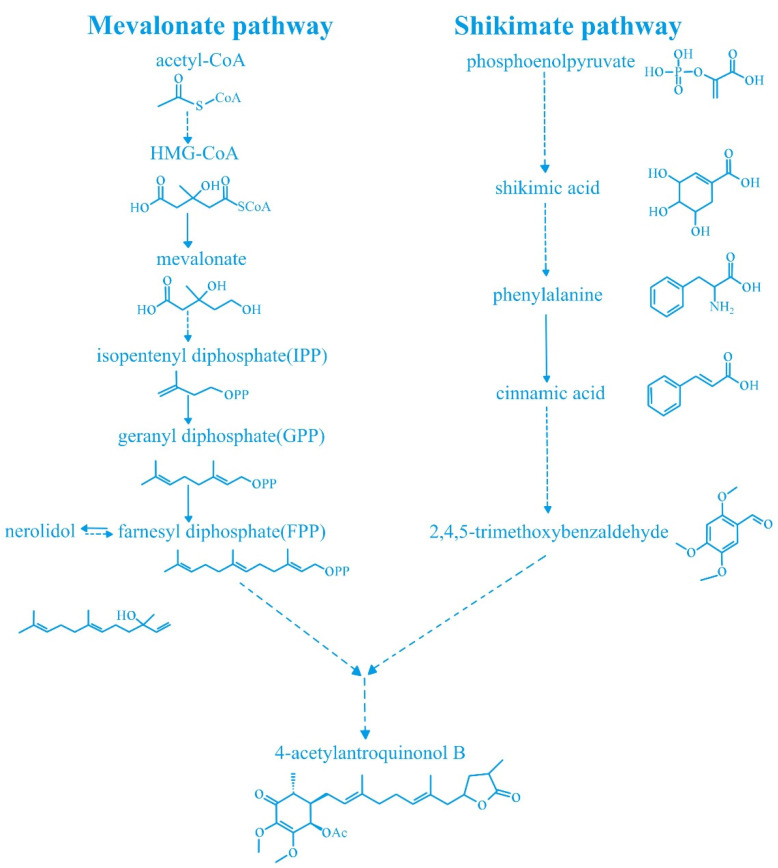
The proposed biosynthetic pathway of 4-acetylarylquinolinol B.

Antrodia cinnamomea is a type of edible fungus found in Taiwan, and is regarded as an important natural resource due to its medicinal and biological properties [1,2]. It has a variety of biological effects, including anticancer, anti-inflammatory, antioxidant, and hepatoprotective activities [3,4,5,6]; therefore, is widely used in the treatment of abdominal pain, inflammation, liver disease, and cancer [3,7]. The compound 4-acetylarylquinolinol B (4-AAQB) (Figure 1) [8,9], one of the main bioactive compounds in the stamens of Antrodia cinnamomea, was isolated and identified in 2009 [9,10]. At present, the biosynthetic pathway of 4-AAQB is not clear, but it may be related to the ubiquinone biosynthetic pathway [11,12,13,14,15]. Figure 2 shows the proposed biosynthetic pathway of 4-AAQB [8]. It has been reported that 4-AAQB has anti-inflammatory, antiproliferative, blood-sugar-lowering, antimetastasis, vascular tone relaxation, and autophagy regulatory activities [7,9,16], and is involved in many kinds of cancers [17]. In this review, we summarized the roles and mechanisms of 4-AAQB in different pathological processes studied in recent years, hoping to provide a theoretical reference for future related research.

## 2. The Protective Role of 4-Acetylarylquinolinol B in Cancer

### 2.1. The Protective Role of 4-Acetylarylquinolinol B in Hepatocellular Carcinoma

Hepatocellular carcinoma (HCC) is a very heterogeneous malignant disease among tumors found so far, and is the second most common cause of cancer death, with more than 700,000 deaths a year [18,19,20]. The main risk factors for HCC are alcohol consumption, hepatitis B/C virus infection, metabolic disorder, and aflatoxin B1 [21]. HCC most often occurs in the case of chronic liver inflammation and fibrosis, and then develops into tumors through a variety of processes [18]. At present, the pathogenesis of HCC is not completely clear; therefore, there is a lack of effective treatments for HCC [22]. The compound 4-AAQB has been reported to improve HCC. The results of the study by Yu-Wei Lin et al. showed that after HepG2 cells were treated with 4-AAQB for 72 h, the proportion of cells in the G1 phase of the cell cycle increased significantly, the proportion of cells in the S phase decreased significantly, and the proportion of cells in the G2/M phase had no notable change, indicating that 4-AAQB inhibited the cell cycle by increasing the number of HepG2 cells in the G1 phase and reducing the number of HepG2 cells in the S phase. The mechanism research revealed that the 4-AAQB treatment decreased the levels of CDK2 and CDK4 and increased the p27 level in a dose-dependent manner. When treated with a low dosage (0.1 μg/mL) of 4-AAQB, the protein expressions of p53 and p21 in HepG2 cells were also increased. However, a higher dosage of 4-AAQB reduced the protein expression of p53 and p21 [23]. CDK2 and CDK4 have been considered as the main regulators of G1 arrest [24,25], while p27 is an inhibitor of CDK2 and CDK4, and can lead to cell cycle arrest at the G1 phase [26,27,28]. Collectively, 4-AAQB inhibited the growth of HepG2 cells in vitro by inducing cell cycle arrest, mostly through p27-mediated decreases in CDK2 and CDK4. In addition, p53 and p21 also played minor roles in the growth inhibition of HepG2 when cells were treated with low dose of 4-AAQB. In the above study, when the cells were treated with 4-AAQB, p27 mRNA decreased, while the p27 protein level increased [23]. Different from traditional tumor-suppressor genes, p27 gene is rarely inactivated in cancer cells in a homozygous manner. Therefore, the reason for the opposite changes in p27 mRNA and protein induced by 4-AAQB is that 4-AAQB upregulates the p27 protein level by reducing its degradation in HepG2 cells [29,30,31]. How 4-AAQB regulates the degradation of p27 needs to be further clarified. The degradation pathway of p27 is a promising target for HCC treatment.

Similar to the above, another study by Chien-Hsin Chang et al. showed that 4-AAQB suppressed the proliferation of hepatoma cells (HepG2 and Huh-7 cells) by inducing cell cycle arrest in the G1 phase in a dose-dependent manner in vitro and in vivo, and significantly inhibited the growth of Huh-7 hepatoma cells in xenotransplantation and in situ models. The mechanism study revealed that 4-AAQB could effectively suppress the phosphorylation of mTOR, PI3K, ERK, and Akt in Huh-7 cells, indicating that the PI3K/Akt/mTOR and ERK pathways were inhibited by 4-AAQB. The treatment of Huh-7 cells with LY294002 (PI3K-specific inhibitor), PD98059 (ERK-specific inhibitor), or rapamycin (mTOR inhibitor) had similar inhibitory effects to those of 4-AAQB on Huh-7 cell proliferation, indicating that 4-AAQB inhibited HCC by suppressing the PI3K/Akt/mTOR and ERK pathways. 4-AAQB significantly reduced the production and release of vascular endothelial growth factor(VEGF), and inhibited the activity of Rho GTPases. In addition, 4-AAQB suppressed Huh-7 cell migration in vitro and its lung metastasis in vivo [9]. The high level of VEGF is involved in the migration of HCC [32] and the activated PI3K/Akt/mTOR pathway could upregulate VEGF production, indicating that PI3K/Akt/mTOR pathway mediated the inhibition of VEGF by 4AAQB [33]. The Rho GTPase signaling cascade, which includes Rho and Rac, is activated by VEGF, and is an important regulator of cell migration [34], indicating that 4-AAQB suppressed Huh-7 cells migration in vitro and its lung metastasis in vivo via Rho GTPase signaling cascade. Collectively, 4-AAQB inhibits hepatoma cells proliferation and metastasis via suppressing PI3K/Akt/mTOR, ERK pathways and inhibition of Rho GTPase signaling pathway via suppressing VEGF production [9]. It has been reported that PI3K/AKT/mTOR promotes G1 progression and inhibits apoptosis in cancers [35,36,37]. However, the above research revealed that 4-AAQB only promoted G1 phase arrest via PI3K/AKT/mTOR pathway, but could not induce apoptosis [9], the reason needs to be clarified. PI3K/Akt/mTOR signaling pathway will become an important target of 4-AAQB in HCC. Whether 4-AAQB plays an anti-HCC role through other signal pathways needs to be further studied.

In addition to inducing cell cycle arrest, 4-AAQB can also improve HCC through cancer stem cells(CSCs). CSCs are a kind of cells with self-renewal and differentiation ability. CSCs can lead to tumor metastatic recurrence and play an important role in the occurrence and development of a variety of tumors [38]. The reason why CSCs inhibits tumor immunity may be that it can lead to tumor immune escape [39]. Moreover, CSCs also can resist apoptosis-inducing immune killing by expressing anti-apoptotic proteins such as Bcl-2 and Bcl-xL, or suppress immune response by secreting immunosuppressive cytokines [40]. The dendritic cells (DCs) vaccines can inhibit CSCs, thus having better therapeutic effects on cancers [41]. Ting Yi Li and colleagues demonstrated that 4-AAQB could suppress liver cancer stem cells(LCSCs) marker, EpCAM and AFP, in a dose-dependent manner and promote the antitumor ability of DCs, indicating that 4-AAQB inhibited LCSC by activating DCs. The compound 4-AAQB also notably suppressed the tumorigenicity by decreasing β-catenin protein expression, and reducing the secretion of immune-escape-related cytokines. Furthermore, 4-AAQB could promote immune-cell proliferation and DC maturation, as well as ameliorate the tumor immune environment of LCSCs, by reducing the immunosuppressive cytokines IL-6 and IL-10. When immature DCs were cocultured with EpCAM + HepG2 cells, 4-AAQB could promote the expression of MHC class I and II on the surfaces of LCSCs and DCs, and upregulate the expression of dendritic cell costimulatory molecule CD80 and cytokines related to immune activation, suggesting that 4-AAQB enhanced the antigen presentation ability of DCs. Thus, 4-AAQB could suppress HCC by inhibiting LCSC via strengthening the immune function of DCs [42]. In recent years, tumor immunotherapy has developed rapidly. It is well known that the function of DCs in cancer patients may be abnormal. Therefore, targeting DCs is an important tumor immunotherapy [43]. IL-6 can inhibit Th1 immunity of tumors [44,45], while 4-AAQB notably inhibited the IL-6 of EpCAM + HepG2 cells at a very low dose [42], indicating that 4-AAQB improved the immune function of DCs via IL-6 inhibition. The aforementioned study indicated that 4-AAQB could strengthen the immune function of DCs against LCSCs by inducing DC maturation and enhancing their antigen presentation ability. Whether 4-AAQB can activate DCs through other mechanisms remains to be further studied.

### 2.2. The Protective Role of 4-Acetylarylquinolinol B in Glioblastoma

Glioblastoma (GBM) is an inherent brain tumor that is considered to originate from glial stem cells or progenitor cells [46]. It is a common primary malignant central nervous system (CNS) tumor with a 5-year survival rate of only 5% and a median survival rate of <15 months [47,48]. GBM has the characteristics of continuous self-renewal potential, strong tumorigenicity and invasiveness, high recurrence, and resistance to chemotherapy. Related to the above characteristics is a small subset of tumor cells of GBM called GBM stem cells (GBM-SCs) [49,50]. The results of the study by Heng-Wei Liu et al. showed that compared with other glioma types, the abnormal expression of β-catenin was a characteristic of GBM and was associated with the poor prognosis in patients with GBM. Immunohistochemistry showed an increased β-catenin expression in primary and recurrent GBM compared with the adjacent non-neoplastic brain tissues, indicating that the aberrant expression of β-catenin was involved in GBM, and was the cause of poor prognosis. The in-depth studies revealed that β-catenin promoted the carcinogenicity and recurrence of GBM, as well as its cancer stem-cell-like characteristics. In human GBM cell lines (U87MG and DBTRG-05MG cells), 4-AAQB notably reduced the β-catenin level and disrupted GBM-SC-associated oncogenic β-catenin/TCF-1/STAT3 signaling in a dose-dependent manner. The decrease in the β-catenin level induced by 4-AAQB was positively correlated with the reductions in β-catenin, Sox2, and Oct4 expression in the nucleus. Moreover, 4-AAQB significantly decreased the viability of U87MG and DBTRG-05MG cells, and effectively inhibited the migration and invasion of GBM cells as evidenced by the downregulation of the levels of catenin, vimentin, and slug [51]. The undifferentiated GBM-SCs have strong proliferation, invasiveness, and drug resistance, as well as high colony and tumor ball formation efficiency, and are related to tumor formation, recurrence, and reduced treatment response [52,53,54]. The compound 4-AAQB can significantly inhibit the stem-cell-like phenotype and reduce the self-renewal ability of GBM cells, which is consistent with the forementioned notable reductions in expressions or in the nuclear localizations of β-catenin, Sox2, and Oct4. The results of tumor xenotransplantation in vivo confirmed the results in vitro, showing that 4-AAQB significantly inhibited tumor growth induced by GBM-SCs in vivo. Summarily, 4-AAQB significantly suppressed GBM-SC-mediated tumorigenesis by inhibiting β-catenin, suggesting that β-catenin of GBM-SCs is a potential target in the treatment of GBM [50]. Previous studies have shown that 4-AAQB could inhibit tumor stem cells by enhancing DCs’ immunity [42]; therefore, whether 4-AAQB can suppress GBM-SCs via DCs needs to be studied.

### 2.3. The Protective Role of 4-Acetylarylquinolinol B in Colorectal Cancer

Colorectal cancer (CRC) has been reported to be the second most commonly diagnosed cancer in the world [55,56]. Although the great progress has been made in the field of the cancer treatment, nearly 86% of patients with end-stage tumors die within 5 years after the initial diagnosis [57]. The incidence rate of CRC varies from region to region, usually associated with a western lifestyle, decreased physical activity, obesity, and poor diet [58,59]. An increasing amount of evidences have showed that tumor stem cells in CRC are associated with therapeutic resistance, tumor regeneration, and recurrence of CRC [60,61,62,63]. It has been reported that superoxide dismutases (SODs) and miRNA are involved in CRC [64,65,66]. The results of the study by Oluwaseun Adebayo Bamodu et al. showed that in CRC, the expression of SOD2 increased and the expression of hsa-mir-324-5p decreased, which were related to the severity of the CRC. Furthermore, hsa-mir-324 could directly interact with and inhibit the expression of SOD2 in CRC cells, while 4-AAQB significantly inhibited the cell viability of the human CRC cell lines DLD1 and HCT116 by weakening their proliferation, migration, invasion, and clonogenic ability, and inhibited the tumorigenicity of CRC. The mechanism of study revealed that 4-AAQB could upregulate hsa-mir-324-5p expression and decrease SOD2 expression, which was associated with the simultaneous downregulation of SOD2, N-cadherin, vimentin, c-myc, and bcl-xl2, accompanied by the upregulation of E-cadherin and the bax2 protein, indicating that 4-AAQB played an anticancer role by inhibiting epithelial mesenchymal transformation (EMT) and promoting apoptosis of CRC cells through hsa-mir-324. The compound 4-AAQB also effectively inhibited the SOD2-promoted CSC-like phenotype, as evidenced by suppressing the proliferation and self-renewal of CRC cells. The upregulation of hsa-mir-324-5p expression in CRC cells inhibited their tumorigenicity in vitro and in vivo. In addition, 4-AAQB synergistically enhanced the anticancer effect of FOLFOX (folinate (folic acid), fluorouracil (5FU) and oxaliplatin) by inducing the re-expression of hsa-mir-324 inhibited by SOD2 and suppressing SOD2-mediated tumorigenicity. Collectively, 4-AAQB suppressed SOD2-enhanced tumorigenicity by promoting hsa-mir-324 expression [67]. SOD2 was previously reported to be a tumor suppressor [68], which was contrary to the results of the above studies. This may have been due to the different expression levels of SOD2 in different tumor tissues. Whether 4-AAQB can inhibit tumors by upregulating SOD2 in some tumors remains to be studied. Additionally, the mechanism of 4-AAQB’s action on hsa-mir-324 remains to be clarified.

## 3. The Protective Role of 4-Acetylarylquinolinol B in Nonalcoholic Fatty Liver Disease

Nonalcoholic fatty liver disease (NAFLD), including fatty liver, nonalcoholic steatohepatitis, and liver cirrhosis, is a clinicopathological syndrome characterized by liver fat accumulation, excluding excessive drinking and viral infection. Due to its high incidence rate (about 20–30%) and the lack of an effective clinical treatment, NAFLD has become a serious chronic disease in the world [52,53,54]. NAFLD is associated with many factors, including type 2 diabetes mellitus (T2DM), insulin resistance, dyslipidemia, and hypertension; however, the exact mechanism is not fully understood [69,70]. I-Chuan Yen and colleagues found that 4-AAQB ameliorated methionine/choline-deficient(MCD) diet-induced NASH by attenuating steatosis, hepatic ballooning, and immune cell filtration, and by reducing the plasma levels of ALT and AST. The mechanism research showed that 4-AAQB suppressed NLRP3 inflammasome-mediated inflammation by reducing the levels of NLRP3, ASC, caspase-1, and IL-1β; mitigated endoplasmic reticulum (ER) stress by suppressing IRE1α, PERK, CHOP, and eIF2α; and activated the nuclear factor erythroid 2-related factor 2 (Nrf2) and Sirtuin 1(SIRT1) signaling pathways in in vitro and in vivo models [71]. Evidence indicated that the ER stress/NLRP3 inflammasome pathway was significantly correlated with NAFLD [72,73], suggesting that 4-AAQB improved NAFLD by inhibiting the ER stress/NLRP3 inflammasome pathway. SIRT1 ameliorated NAFLD by inhibiting hepatic inflammation, ER stress, and lipogenesis [74,75,76]. Contrarily, SIRT1 gene knockout exacerbated NAFLD by activating the NLRP3 inflammasome and subsequent inflammation, indicating that SIRT1 mediated 4-AAQB improvement of NAFLD via suppressing the ER stress/NLRP3 inflammasome [77]. The research has revealed that Nrf2-deficient mice were prone to NASH [78]. Therefore, Nrf2 is also regarded as a promising therapeutic target for NAFLD [79,80]. Furthermore, activation of Nrf2 ameliorated NASH progression by suppressing ER stress [81]. SIRT1 was involved in the activation of Nrf2 in vivo [82]. All in all, it was indicated that 4-AAQB ameliorated NAFLD by inhibiting the ER stress/NLRP3 inflammasome by activating the SIRT1-Nrf2 pathway, which needs to be further confirmed [71]. Our previous studies showed that the autophagy/NLRP3 inflammasome was closely related to liver lipid metabolism [83,84], and 4-AAQB could regulate autophagy [85]. These indicated that whether 4-AAQB can improve NAFLD by regulating the autophagy/NLRP3 inflammasome is worthy of further study.

## 4. The Protective Role of 4-Acetylarylquinolinol B in Inflammation

The inflammation is a protective reaction of the body that is triggered by the noxious stimuli such as infection and tissue injury [86,87,88]. The invading pathogens activate macrophages to release proinflammatory cytokines (tumor necrosis factor (TNF)-α, interleukin (IL)-6, and IL-1 β) and proinflammatory mediators such as cyclooxygenase (COX)-2 and nitric oxide (NO) [89]. Sepsis is a serious systemic inflammatory response and immune-system response pattern to injury that eventually leads to multiple organ dysfunction [90,91]. The compound 4-AAQB has been reported to have anti-inflammatory effects [6]. Chien Hsin Chang and colleagues found that 4-AAQB inhibited the production of TNF-α/IL-6 in lipopolysaccharide (LPS)-induced RAW264.7 macrophages and peritoneal macrophages, and LPS-induced peritoneal macrophage migration in a dose-dependent manner. In LPS-induced macrophages, 4-AAQB could also suppress the expression of the inducible nitric oxide synthase (iNOS) and NO production. The aforementioned indicated that 4-AAQB could inhibit the production of proinflammatory mediators induced by LPS. In LPS-induced macrophages, the phosphorylation levels of extracellular signal related kinase 1/2 (ERK1/2), p38 MAP kinase (p38), and c-jun NH2 terminal kinase (JNK) were upregulated, while 4-AAQB could inhibit this change, indicating that 4-AAQB suppressed the MAPK pathway that was involved in inflammation. The compound 4-AAQB also inhibited the phosphorylation of NF-κB subunit p65 and IkBα, as well as STAT1. All in all, 4-AAQB inhibited LPS-induced inflammation through the inhibition of MAPK, NF-κB, and STAT-1, and is a candidate for the treatment of inflammatory diseases [92]. In the above studies, 4-AAQB inhibited the production and release of proinflammatory factors through the MAPK, STAT1, and NF-KB pathways. Whether 4-AAQB can promote the production and release of anti-inflammatory factors through some signal pathways is a topic worthy of study. The NLRP3 inflammasome plays an important role in inflammation [83,84]. Therefore, whether 4-AAQB can inhibit NLRP3-inflammasome-mediated inflammation remains to be clarified.

## 5. The Protective Role of 4-Acetylarylquinolinol B in Osteoclastogenesis

Studies have revealed that the exposure to a microgravity (µXg) environment can lead to reduced osteoblast formation and osteoclast differentiation induction; thus, the bone loss remains a problem for astronauts [93,94]. Therefore, the inhibition of osteoclastogenesis is considered to be a promising strategy for the prevention of osteoporosis under µXg conditions [94,95]. Chia-Hsin Wu et al. exposed the monocyte/macrophage-like cell line RAW264 7 to simulated µXg for 24 h, followed by treatment with 4-AAQB/alendronate (AlN) and osteoclast differentiation factor receptor activator of nuclear factor kappa-B ligand (RANKL). The results showed that 4-AAQB weakened osteoclast formation and reabsorption induced by RANKL under µXg conditions, and that 4-AAQB also notably inhibited µXg-induced osteoclast differentiation, as evidenced by the reduction in the critical regulators of osteoclast differentiation, including c-Fos, nuclear factor of activated T-cells cytoplasmic 1 (NFATc1), and dendritic cell-specific transmembrane protein (DC-STAMP). The in-depth research revealed that 4-AAQB upregulated the levels of cleaved caspase-3 and cleaved PARP, but downregulated cleaved caspase-8 levels in µXg. Moreover, the protein levels of cyclin E, cyclin D3, and p21 were reduced by 4-AAQB. These results indicated that 4-AAQB promoted apoptosis and caused cell cycle arrest at the G1-S phase under µXg conditions. Meanwhile, 4-AAQB also significantly inhibited autophagy by reducing the levels of LC3B-II/LC3B-I and p62. While 3-methyladenine (3-MA) and chloroquine (CQ) (two autophagy inhibitors) had similar effects to those of 4-AAQB, indicating that 4-AAQB suppressed RANKL-induced osteoclastogenesis by suppressing autophagy under µXg conditions, and that 4-AAQB can be used as a potential agent against µXg-induced osteoclast formation [96]. Coenzyme Q10, which has a similar chemical structure to 4-AAQB, could inhibit osteoclast differentiation and promote the proliferation and differentiation of osteoblasts [97,98,99]. Whether 4-AAQB can promote the proliferation and differentiation of osteoblasts is worthy of further study.

## 6. Conclusions

**Figure 3:**
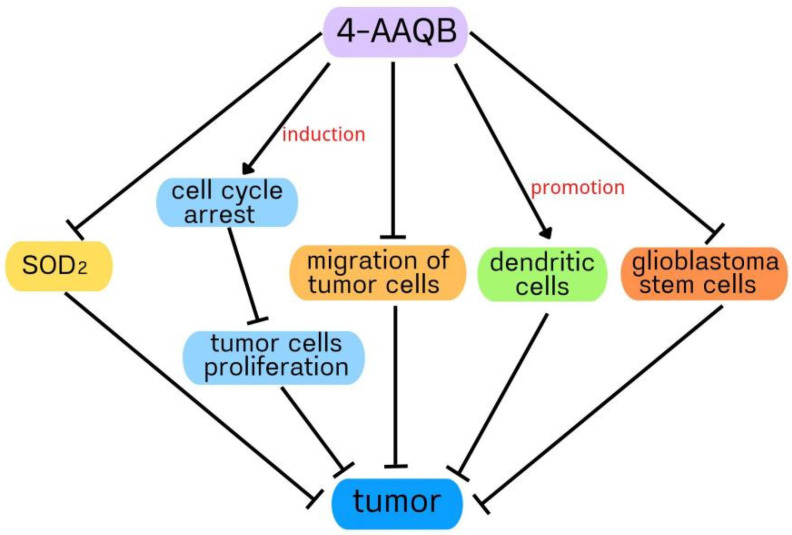
Schematic diagram of the antitumor mechanism of 4-AAQB.

Recent studies have shown that 4-AAQB plays a protective role in many pathological processes. In this review, we summarized these as follows: (1) 4-AAQB inhibited HepG2 cells proliferation in vitro through induction of cell cycle arrest, mostly via p27-mediated decrease of CDK2 and CDK4; (2) 4-AAQB suppressed hepatoma cell proliferation and metastasis by inhibiting the PI3K/Akt/mTOR and ERK pathways, as well as VEGF production; (3) 4-AAQB could suppress HCC by suppressing LCSC via strengthening of the immune function of dendritic cells; (4) 4-AAQB notably inhibited GBM-SC-mediated tumorigenesis through β-catenin inhibition; (5) 4-AAQB ameliorated NAFLD through inhibition of the ER stress/NLRP3 inflammasome by activating the SIRT1-Nrf2 pathway, which requires further confirmation; (6) 4-AAQB improved CRC by inhibiting SOD2-enhanced tumorigenicity via the promotion of hsa-mir-324 expression; (7) 4-AAQB inhibited LPS-induced inflammation and mitigated sepsis by suppressing the MAPK, STAT1, and NF-kB pathways; and (8) 4-AAQB improved RANKL-induced osteoclastogenesis by inhibiting autophagy under µXg conditions (Table 1). It can be seen from the above that 4-AAQB can play an antitumor role by regulating the cell cycle, cell migration, SOD2, cancer stem cells, and cellular immunity (Figure 3). Whether the dosage of 4-AAQB is related to its antitumor effect and whether its antitumor effect has side effects need to be further studied. It can be seen from the above that 4-AAQB can promote lipid metabolism and inhibit inflammation via NLRP3 inflammasome/ER stress/autophagy. indicting that the NLRP3 inflammasome, endoplasmic reticulum stress, and autophagy are common important targets of 4-AAQB in different diseases. Therefore, the improvement of 4-AAQB in targeting the NLRP3 inflammasome/ER stress/autophagy in different diseases is a topic worthy of study in the future. Further, the signal pathways involved in the action of 4-AAQB need to be further studied. The synthesis and metabolism of 4-AAQB will be the focus of future research.

It is believed that after an in-depth study of its biological function, 4-AAQB will become an important drug for the treatment of related diseases.

## Figures and Tables

**Table 1:** Summary of protective roles of 4-acetylarylquinolinol B in pathological processes.

Type of Pathological Processes	Protective Mechanism of 4-Acetylarylquinolinol B	Experimental Model	Reference
Liver cancer	Induction of cell cycle arrest, mostly via p27-mediated decreases in CDK2 and CDK4	HepG2 cells	[23]
Liver cancer	Inhibition of PI3K/Akt/mTOR and ERK pathways, as well as VEGF production	HepG2 and Huh-7	[9]
Liver cancer	Inhibition of LCSC via strengthening of the immune function of dendritic cells	HepG2 cells model	[42]
Glioblastoma (GBM)	Inhibition of GBM stem-cell-mediated tumorigenesis by suppressing β-catenin	Human GBM cell lines U87MG and DBTRG-05MG	[50]
Nonalcoholic fatty liver diseases (NAFLD)	Inhibition of the ERS/NLRP3 inflammasome by activating the SIRT1-Nrf2 pathway	C57BL/6 mice, NAFLD model	[71]
Colorectal cancer (CRC)	Inhibition of SOD2-enhanced tumorigenicity via promotion of hsa-mir-324 expression	Human CRC cell lines DLD-1 and HCT116	[67]
Inflammation	Inhibition of MAPK, STAT1, and NF-kB pathways	Murine macrophages, peritoneal macrophages, and mice	[92]
Osteoclastogenesis	Inhibition of autophagy under µXg Conditions	RAW264.7 cell line	[96]

## References

[1] Chen S.Y., Lee Y.R., Hsieh M.C., Omar H.A., Teng Y.N., Lin C.Y., Hung J.H. (2018). Enhancing the Anticancer Activity of *Antrodia cinnamomea* in Hepatocellular Carcinoma Cells via Cocultivation with Ginger: The Impact on Cancer Cell Survival Pathways. Front. Pharmacol..

[2] Huang H.T., Wang S.L., Nguyen V.B., Kuo Y.H. (2018). Isolation and Identification of Potent Antidiabetic Compounds from *Antrodia cinnamomea*—An Edible Taiwanese Mushroom. Molecules.

[3] Ao Z.H., Xu Z.H., Lu Z.M., Xu H.Y., Zhang X.M., Dou W.F. (2009). Niuchangchih (*Antrodia camphorata*) and its potential in treating liver diseases. J. Ethnopharmacol..

[4] Geethangili M., Tzeng Y.M. (2011). Review of Pharmacological Effects of *Antrodia camphorata* and Its Bioactive Compounds. Evid.-Based Complement. Altern. Med..

[5] Hsiao G., Shen M.Y., Lin K.H., Lan M.H., Wu L.Y., Chou D.S., Lin C.H., Su C.H., Sheu J.R. (2003). Antioxidative and hepatoprotective effects of *Antrodia camphorata* extract. J. Agric. Food Chem..

[6] Wen C.L., Chang C.C., Huang S.S., Kuo C.L., Hsu S.L., Deng J.S., Huang G.J. (2011). Anti-inflammatory effects of methanol extract of *Antrodia cinnamomea* mycelia both in vitro and in vivo. J. Ethnopharmacol..

[7] Lu M.C., El-Shazly M., Wu T.Y., Du Y.C., Chang T.T., Chen C.F., Hsu Y.M., Lai K.H., Chiu C.P., Chang F.R. (2013). Recent research and development of *Antrodia cinnamomea*. Pharmacol. Ther..

[8] Chiang C.C., Huang T.N., Lin Y.W., Chen K.H., Chiang B.H. (2013). Enhancement of 4-acetylantroquinonol B production by supplementation of its precursor during submerged fermentation of *Antrodia cinnamomea*. J. Agric. Food Chem..

[9] Chang C.H., Huang T.F., Lin K.T., Hsu C.C., Chang W.L., Wang S.W., Ko F.N., Peng H.C., Chung C.H. (2015). 4-Acetylantroquinonol B suppresses tumor growth and metastasis of hepatoma cells via blockade of translation-dependent signaling pathway and VEGF production. J. Agric. Food Chem..

[10] Yang S.S., Wang G.J., Wang S.Y., Lin Y.Y., Kuo Y.H., Lee T.H. (2009). New constituents with iNOS inhibitory activity from mycelium of *Antrodia camphorata*. Planta Med..

[11] Bentinger M., Tekle M., Dallner G. (2010). Coenzyme Q—Biosynthesis and functions. Biochem. Biophys. Res. Commun..

[12] Knaggs A.R. (2003). The biosynthesis of shikimate metabolites. Nat. Prod. Rep..

[13] Nierop Groot M.N., de Bont J.A.M. (1998). Conversion of phenylalanine to benzaldehyde initiated by an aminotransferase in *Lactobacillus plantarum*. Appl. Environ. Microbiol..

[14] Roberts C.W., Roberts F., Lyons R.E., Kirisits M.J., Mui E.J., Finnerty J., Johnson J.J., Ferguson D.J., Coggins J.R., Krell T. (2002). The shikimate pathway and its branches in apicomplexan parasites. J. Infect. Dis..

[15] Ladygina N., Dedyukhina E.G., Vainshtein M.B. (2006). A review on microbial synthesis of hydrocarbons. Process Biochem..

[16] Chang T.C., Yeh C.T., Adebayo B.O., Lin Y.C., Deng L., Rao Y.K., Huang C.C., Lee W.H., Wu A.T., Hsiao M. (2015). 4-Acetylantroquinonol B inhibits colorectal cancer tumorigenesis and suppresses cancer stem-like phenotype. Toxicol. Appl. Pharmacol..

[17] Lin T.C., Germagian A., Liu Z. (2021). The NF-*κ*B Signaling and Wnt/*β*-catenin Signaling in MCF-7 Breast Cancer Cells in Response to Bioactive Components from Mushroom *Antrodia camphorata*. Am. J. Chin. Med..

[18] Li L., Wang H. (2016). Heterogeneity of liver cancer and personalized therapy. Cancer Lett..

[19] Sugawara Y., Hibi T. (2021). Surgical treatment of hepatocellular carcinoma. Biosci. Trends.

[20] El-Khoueiry A.B., Hanna D.L., Llovet J., Kelley R.K. (2021). Cabozantinib: An evolving therapy for hepatocellular carcinoma. Cancer Treat. Rev..

[21] Chatterjee R., Mitra A. (2015). An overview of effective therapies and recent advances in biomarkers for chronic liver diseases and associated liver cancer. Int. Immunopharmacol..

[22] Wang H., Lu Z., Zhao X. (2019). Tumorigenesis, diagnosis, and therapeutic potential of exosomes in liver cancer. J. Hematol. Oncol..

[23] Lin Y.W., Chiang B.H. (2011). 4-acetylantroquinonol B isolated from *Antrodia cinnamomea* arrests proliferation of human hepatocellular carcinoma HepG2 cell by affecting p53, p21 and p27 levels. J. Agric. Food Chem..

[24] Tan G., Zhang G.Y., Xu J., Kang C.W., Yan Z.K., Lei M., Pu X.B., Dong C.C. (2020). PLA2G10 facilitates the cell-cycle progression of soft tissue leiomyosarcoma cells at least by elevating cyclin E1/CDK2 expression. Biochem. Biophys. Res. Commun..

[25] Xu L., Zhang X., Xiao S., Li X., Jiang H., Wang Z., Sun B., Zhao Y. (2021). Panaxadiol as a major metabolite of AD-1 can significantly inhibit the proliferation and migration of breast cancer cells: In vitro and in vivo study. Bioorg. Chem..

[26] Cho A.R., Park W.Y., Lee H.J., Sim D.Y., Im E., Park J.E., Ahn C.H., Shim B.S., Kim S.H. (2021). Antitumor Effect of Morusin via G1 Arrest and Antiglycolysis by AMPK Activation in Hepatocellular Cancer. Int. J. Mol. Sci..

[27] Dutta N., Pemmaraju D.B., Ghosh S., Ali A., Mondal A., Majumder C., Nelson V.K., Mandal S.C., Misra A.K., Rengan A.K. (2022). Alkaloid-rich fraction of *Ervatamia coronaria* sensitizes colorectal cancer through modulating AMPK and mTOR signalling pathways. J. Ethnopharmacol..

[28] Oliveira H.A., Bueno A.C., Pugliesi R.S., da Silva Junior R.M.P., de Castro M., Martins C.S. (2022). PI3K inhibition by BKM120 results in anti-proliferative effects on corticotroph tumor cells. J. Endocrinol. Investig..

[29] Kawamata N., Morosetti R., Miller C.W., Park D., Spirin K.S., Nakamaki T., Takeuchi S., Hatta Y., Simpson J., Wilcyznski S. (1995). Molecular analysis of the cyclin-dependent kinase inhibitor gene p27/Kip1 in human malignancies. Cancer Res..

[30] Ponce-Castaneda M.V., Lee M.H., Latres E., Polyak K., Lacombe L., Montgomery K., Mathew S., Krauter K., Sheinfeld J., Massague J. (1995). p27Kip1: Chromosomal mapping to 12p12-12p13.1 and absence of mutations in human tumors. Cancer Res..

[31] Pietenpol J.A., Bohlander S.K., Sato Y., Papadopoulos N., Liu B., Friedman C., Trask B.J., Roberts J.M., Kinzler K.W., Rowley J.D. (1995). Assignment of the human p27Kip1 gene to 12p13 and its analysis in leukemias. Cancer Res..

[32] Song J., Guan Z., Song C., Li M., Gao Z., Zhao Y. (2021). Apatinib suppresses the migration, invasion and angiogenesis of hepatocellular carcinoma cells by blocking VEGF and PI3K/AKT signaling pathways. Mol. Med. Rep..

[33] Chen J., Zhang X., Liu X., Zhang C., Shang W., Xue J., Chen R., Xing Y., Song D., Xu R. (2019). Ginsenoside Rg1 promotes cerebral angiogenesis via the PI3K/Akt/mTOR signaling pathway in ischemic mice. Eur. J. Pharmacol..

[34] Chen Z.L., Yang J., Shen Y.W., Li S.T., Wang X., Lv M., Wang B.Y., Li P., Zhao W., Qiu R.Y. (2018). AmotP130 regulates Rho GTPase and decreases breast cancer cell mobility. J. Cell. Mol. Med..

[35] Duan S., Huang W., Liu X., Liu X., Chen N., Xu Q., Hu Y., Song W., Zhou J. (2018). IMPDH2 promotes colorectal cancer progression through activation of the PI3K/AKT/mTOR and PI3K/AKT/FOXO1 signaling pathways. J. Exp. Clin. Cancer Res..

[36] Yang J., Pi C., Wang G. (2018). Inhibition of PI3K/Akt/mTOR pathway by apigenin induces apoptosis and autophagy in hepatocellular carcinoma cells. Biomed. Pharmacother..

[37] Zhou J., Jiang Y.Y., Chen H., Wu Y.C., Zhang L. (2020). Tanshinone I attenuates the malignant biological properties of ovarian cancer by inducing apoptosis and autophagy via the inactivation of PI3K/AKT/mTOR pathway. Cell Prolif..

[38] Recalcati S., Gammella E., Cairo G. (2019). Dysregulation of iron metabolism in cancer stem cells. Free Radic. Biol. Med..

[39] Bruttel V.S., Wischhusen J. (2014). Cancer stem cell immunology: Key to understanding tumorigenesis and tumor immune escape?. Front. Immunol..

[40] Todaro M., Alea M.P., Di Stefano A.B., Cammareri P., Vermeulen L., Iovino F., Tripodo C., Russo A., Gulotta G., Medema J.P. (2007). Colon cancer stem cells dictate tumor growth and resist cell death by production of interleukin-4. Cell Stem Cell.

[41] Akbulut H., Babahan C., Abgarmi S.A., Ocal M., Besler M. (2019). Recent Advances in Cancer Stem Cell Targeted Therapy. Crit. Rev. Oncog..

[42] Li T.Y., Chiang B.H. (2019). 4-Acetylantroquinonol B from *Antrodia cinnamomea* enhances immune function of dendritic cells against liver cancer stem cells. Biomed. Pharmacother..

[43] Giorello M.B., Matas A., Marenco P., Davies K.M., Borzone F.R., Calcagno M.L., Garcia-Rivello H., Wernicke A., Martinez L.M., Labovsky V. (2021). CD1a- and CD83-positive dendritic cells as prognostic markers of metastasis development in early breast cancer patients. Breast Cancer.

[44] Tsukamoto H., Fujieda K., Senju S., Ikeda T., Oshiumi H., Nishimura Y. (2018). Immune-suppressive effects of interleukin-6 on T-cell-mediated anti-tumor immunity. Cancer Sci..

[45] Brede K.M., Schmid J., Steinmetz O.M., Panzer U., Klinge S., Mittrucker H.W. (2021). Neutralization of IL-6 inhibits formation of autoreactive TH17 cells but does not prevent loss of renal function in experimental autoimmune glomerulonephritis. Immunol. Lett..

[46] Hernandez A., Domenech M., Munoz-Marmol A.M., Carrato C., Balana C. (2021). Glioblastoma: Relationship between Metabolism and Immunosuppressive Microenvironment. Cells.

[47] Stupp R., Hegi M.E., Mason W.P., van den Bent M.J., Taphoorn M.J., Janzer R.C., Ludwin S.K., Allgeier A., Fisher B., Belanger K. (2009). Effects of radiotherapy with concomitant and adjuvant temozolomide versus radiotherapy alone on survival in glioblastoma in a randomised phase III study: 5-year analysis of the EORTC-NCIC trial. Lancet Oncol..

[48] Ostrom Q.T., Gittleman H., Fulop J., Liu M., Blanda R., Kromer C., Wolinsky Y., Kruchko C., Barnholtz-Sloan J.S. (2015). CBTRUS Statistical Report: Primary Brain and Central Nervous System Tumors Diagnosed in the United States in 2008–2012. Neuro-Oncol..

[49] Gimple R.C., Bhargava S., Dixit D., Rich J.N. (2019). Glioblastoma stem cells: Lessons from the tumor hierarchy in a lethal cancer. Genes Dev..

[50] Stupp R., Mason W.P., van den Bent M.J., Weller M., Fisher B., Taphoorn M.J., Belanger K., Brandes A.A., Marosi C., Bogdahn U. (2005). Radiotherapy plus concomitant and adjuvant temozolomide for glioblastoma. N. Engl. J. Med..

[51] Liu H.W., Su Y.K., Bamodu O.A., Hueng D.Y., Lee W.H., Huang C.C., Deng L., Hsiao M., Chien M.H., Yeh C.T. (2018). The Disruption of the beta-Catenin/TCF-1/STAT3 Signaling Axis by 4-Acetylantroquinonol B Inhibits the Tumorigenesis and Cancer Stem-Cell-Like Properties of Glioblastoma Cells, In Vitro and In Vivo. Cancers.

[52] Bradshaw A., Wickremsekera A., Tan S.T., Peng L., Davis P.F., Itinteang T. (2016). Cancer Stem Cell Hierarchy in Glioblastoma Multiforme. Front. Surg..

[53] Iacopino F., Angelucci C., Piacentini R., Biamonte F., Mangiola A., Maira G., Grassi C., Sica G. (2014). Isolation of cancer stem cells from three human glioblastoma cell lines: Characterization of two selected clones. PLoS ONE.

[54] Beier D., Schulz J.B., Beier C.P. (2011). Chemoresistance of glioblastoma cancer stem cells—Much more complex than expected. Mol. Cancer.

[55] Arnold M., Sierra M.S., Laversanne M., Soerjomataram I., Jemal A., Bray F. (2017). Global patterns and trends in colorectal cancer incidence and mortality. Gut.

[56] Ciardiello D., Vitiello P.P., Cardone C., Martini G., Troiani T., Martinelli E., Ciardiello F. (2019). Immunotherapy of colorectal cancer: Challenges for therapeutic efficacy. Cancer Treat. Rev..

[57] Siegel R.L., Miller K.D., Fedewa S.A., Ahnen D.J., Meester R.G.S., Barzi A., Jemal A. (2017). Colorectal cancer statistics, 2017. CA Cancer J. Clin..

[58] Dekker E., Tanis P.J., Vleugels J.L.A., Kasi P.M., Wallace M.B. (2019). Colorectal cancer. Lancet.

[59] Keum N., Giovannucci E. (2019). Global burden of colorectal cancer: Emerging trends, risk factors and prevention strategies. Nat. Rev. Gastroenterol. Hepatol..

[60] Colak S., Zimberlin C.D., Fessler E., Hogdal L., Prasetyanti P.R., Grandela C.M., Letai A., Medema J.P. (2014). Decreased mitochondrial priming determines chemoresistance of colon cancer stem cells. Cell Death Differ..

[61] Dylla S.J., Beviglia L., Park I.K., Chartier C., Raval J., Ngan L., Pickell K., Aguilar J., Lazetic S., Smith-Berdan S. (2008). Colorectal cancer stem cells are enriched in xenogeneic tumors following chemotherapy. PLoS ONE.

[62] Lombardo Y., Scopelliti A., Cammareri P., Todaro M., Iovino F., Ricci-Vitiani L., Gulotta G., Dieli F., de Maria R., Stassi G. (2011). Bone morphogenetic protein 4 induces differentiation of colorectal cancer stem cells and increases their response to chemotherapy in mice. Gastroenterology.

[63] Chen B., Zhang D., Kuai J., Cheng M., Fang X., Li G. (2017). Upregulation of miR-199a/b contributes to cisplatin resistance via Wnt/beta-catenin-ABCG2 signaling pathway in ALDHA1(+) colorectal cancer stem cells. Tumor Biol..

[64] Warsinggih, Irawan B., Labeda I., Lusikooy R.E., Sampetoding S., Kusuma M.I., Uwuratuw J.A., Syarifuddin E., Prihantono, Faruk M. (2020). Association of superoxide dismutase enzyme with staging and grade of differentiation colorectal cancer: A cross-sectional study. Ann. Med. Surg..

[65] Xu J., Meng Q., Li X., Yang H., Xu J., Gao N., Sun H., Wu S., Familiari G., Relucenti M. (2019). Long Noncoding RNA MIR17HG Promotes Colorectal Cancer Progression via miR-17-5p. Cancer Res..

[66] Yang Y., Qu A., Wu Q., Zhang X., Wang L., Li C., Dong Z., Du L., Wang C. (2020). Prognostic value of a hypoxia-related microRNA signature in patients with colorectal cancer. Aging.

[67] Bamodu O.A., Yang C.K., Cheng W.H., Tzeng D.T.W., Kuo K.T., Huang C.C., Deng L., Hsiao M., Lee W.H., Yeh C.T. (2018). 4-Acetyl-Antroquinonol B Suppresses SOD2-Enhanced Cancer Stem Cell-Like Phenotypes and Chemoresistance of Colorectal Cancer Cells by Inducing hsa-miR-324 re-Expression. Cancers.

[68] Bravard A., Sabatier L., Hoffschir F., Ricoul M., Luccioni C., Dutrillaux B. (1992). SOD2: A new type of tumor-suppressor gene?. Int. J. Cancer.

[69] Santhekadur P.K., Kumar D.P., Sanyal A.J. (2018). Preclinical models of non-alcoholic fatty liver disease. J. Hepatol..

[70] Shen X., Jin C., Wu Y., Zhang Y., Wang X., Huang W., Li J., Wu S., Gao X. (2019). Prospective study of perceived dietary salt intake and the risk of non-alcoholic fatty liver disease. J. Hum. Nutr. Diet.

[71] Yen I.C., Tu Q.W., Chang T.C., Lin P.H., Li Y.F., Lee S.Y. (2021). 4-Acetylantroquinonol B ameliorates nonalcoholic steatohepatitis by suppression of ER stress and NLRP3 inflammasome activation. Biomed. Pharmacother..

[72] Lebeaupin C., Vallee D., Hazari Y., Hetz C., Chevet E., Bailly-Maitre B. (2018). Endoplasmic reticulum stress signalling and the pathogenesis of non-alcoholic fatty liver disease. J. Hepatol..

[73] Thomas H. (2017). NAFLD: A critical role for the NLRP3 inflammasome in NASH. Nat. Rev. Gastroenterol. Hepatol..

[74] Yoshizaki T., Schenk S., Imamura T., Babendure J.L., Sonoda N., Bae E.J., Oh D.Y., Lu M., Milne J.C., Westphal C. (2010). SIRT1 inhibits inflammatory pathways in macrophages and modulates insulin sensitivity. Am. J. Physiol. Endocrinol. Metab..

[75] Ding R.B., Bao J., Deng C.X. (2017). Emerging roles of SIRT1 in fatty liver diseases. Int. J. Biol. Sci..

[76] Li Y., Xu S., Giles A., Nakamura K., Lee J.W., Hou X., Donmez G., Li J., Luo Z., Walsh K. (2011). Hepatic overexpression of SIRT1 in mice attenuates endoplasmic reticulum stress and insulin resistance in the liver. FASEB J..

[77] Peng Z., Li X., Xing D., Du X., Wang Z., Liu G., Li X. (2018). Nobiletin alleviates palmitic acidinduced NLRP3 inflammasome activation in a sirtuin 1dependent manner in AML12 cells. Mol. Med. Rep..

[78] Chowdhry S., Nazmy M.H., Meakin P.J., Dinkova-Kostova A.T., Walsh S.V., Tsujita T., Dillon J.F., Ashford M.L., Hayes J.D. (2010). Loss of Nrf2 markedly exacerbates nonalcoholic steatohepatitis. Free Radic. Biol. Med..

[79] Du J., Zhang M., Lu J., Zhang X., Xiong Q., Xu Y., Bao Y., Jia W. (2016). Osteocalcin improves nonalcoholic fatty liver disease in mice through activation of Nrf2 and inhibition of JNK. Endocrine.

[80] Chambel S.S., Santos-Goncalves A., Duarte T.L. (2015). The Dual Role of Nrf2 in Nonalcoholic Fatty Liver Disease: Regulation of Antioxidant Defenses and Hepatic Lipid Metabolism. BioMed Res. Int..

[81] Sharma R.S., Harrison D.J., Kisielewski D., Cassidy D.M., McNeilly A.D., Gallagher J.R., Walsh S.V., Honda T., McCrimmon R.J., Dinkova-Kostova A.T. (2018). Experimental Nonalcoholic Steatohepatitis and Liver Fibrosis Are Ameliorated by Pharmacologic Activation of Nrf2 (NF-E2 p45-Related Factor 2). Cell. Mol. Gastroenterol. Hepatol..

[82] Ding Y.W., Zhao G.J., Li X.L., Hong G.L., Li M.F., Qiu Q.M., Wu B., Lu Z.Q. (2016). SIRT1 exerts protective effects against paraquat-induced injury in mouse type II alveolar epithelial cells by deacetylating NRF2 in vitro. Int. J. Mol. Med..

[83] Wang H., Zhong P., Sun L. (2019). Exogenous hydrogen sulfide mitigates NLRP3 inflammasome-mediated inflammation through promoting autophagy via the AMPK-mTOR pathway. Biol. Open.

[84] Wu D., Zhong P., Wang J., Wang H. (2019). Exogenous hydrogen sulfide mitigates LPS + ATP-induced inflammation by inhibiting NLRP3 inflammasome activation and promoting autophagy in L02 cells. Mol. Cell. Biochem..

[85] Liu M., Bamodu O.A., Huang W.C., Zucha M.A., Lin Y.K., Wu A.T.H., Huang C.C., Lee W.H., Yuan C.C., Hsiao M. (2017). 4-Acetylantroquinonol B suppresses autophagic flux and improves cisplatin sensitivity in highly aggressive epithelial cancer through the PI3K/Akt/mTOR/p70S6K signaling pathway. Toxicol. Appl. Pharmacol..

[86] Kuprash D.V., Nedospasov S.A. (2016). Molecular and Cellular Mechanisms of Inflammation. Biochemistry.

[87] Medzhitov R. (2008). Origin and physiological roles of inflammation. Nature.

[88] Gaestel M., Kotlyarov A., Kracht M. (2009). Targeting innate immunity protein kinase signalling in inflammation. Nat. Rev. Drug Discov..

[89] Kirkpatrick B., Miller B.J. (2013). Inflammation and schizophrenia. Schizophr. Bull..

[90] Salomao R., Ferreira B.L., Salomao M.C., Santos S.S., Azevedo L.C.P., Brunialti M.K.C. (2019). Sepsis: Evolving concepts and challenges. Braz. J. Med. Biol. Res..

[91] Faix J.D. (2013). Biomarkers of sepsis. Crit. Rev. Clin. Lab. Sci..

[92] Chang C.H., Hsu C.C., Lee A.S., Wang S.W., Lin K.T., Chang W.L., Peng H.C., Huang W.C., Chung C.H. (2018). 4-Acetylantroquinonol B inhibits lipopolysaccharide-induced cytokine release and alleviates sepsis through of MAPK and NFkappaB suppression. BMC Complement. Altern. Med..

[93] Ontiveros C., McCabe L.R. (2003). Simulated microgravity suppresses osteoblast phenotype, Runx2 levels and AP-1 transactivation. J. Cell. Biochem..

[94] Ethiraj P., Link J.R., Sinkway J.M., Brown G.D., Parler W.A., Reddy S.V. (2018). Microgravity modulation of syncytin-A expression enhance osteoclast formation. J. Cell. Biochem..

[95] Shanmugarajan S., Zhang Y., Moreno-Villanueva M., Clanton R., Rohde L.H., Ramesh G.T., Sibonga J.D., Wu H. (2017). Combined Effects of Simulated Microgravity and Radiation Exposure on Osteoclast Cell Fusion. Int. J. Mol. Sci..

[96] Wu C.H., Ou C.H., Yen I.C., Lee S.Y. (2020). 4-Acetylantroquinonol B Inhibits Osteoclastogenesis by Inhibiting the Autophagy Pathway in a Simulated Microgravity Model. Int. J. Mol. Sci..

[97] Ikegame M., Hattori A., Tabata M.J., Kitamura K.I., Tabuchi Y., Furusawa Y., Maruyama Y., Yamamoto T., Sekiguchi T., Matsuoka R. (2019). Melatonin is a potential drug for the prevention of bone loss during space flight. J. Pineal Res..

[98] Moon H.J., Ko W.K., Jung M.S., Kim J.H., Lee W.J., Park K.S., Heo J.K., Bang J.B., Kwon I.K. (2013). Coenzyme q10 regulates osteoclast and osteoblast differentiation. J. Food Sci..

[99] Zheng D., Cui C., Yu M., Li X., Wang L., Chen X., Lin Y. (2018). Coenzyme Q10 promotes osteoblast proliferation and differentiation and protects against ovariectomy-induced osteoporosis. Mol. Med. Rep..

